# Dietary Acid Load Modulation of Asthma-Related miRNAs in the Exhaled Breath Condensate of Children

**DOI:** 10.3390/nu14061147

**Published:** 2022-03-08

**Authors:** Francisca Castro Mendes, Inês Paciência, João Cavaleiro Rufo, Diana Silva, Luís Delgado, André Moreira, Pedro Moreira

**Affiliations:** 1 Serviço de Imunologia Basica e Clinica, Departamento de Patologia, Faculdade de Medicina da Universidade do Porto, 4200-319 Porto, Portugal; francisca_castromendes@hotmail.com (F.C.M.); disolha@gmail.com (D.S.); ldelgado@med.up.pt (L.D.); andremoreira@med.up.pt (A.M.); 2 EPIUnit—Instituto de Saude Publica, Universidade do Porto, 4200-450 Porto, Portugal; inespaciencia@gmail.com (I.P.); jcrufo@gmail.com (J.C.R.); 3 Laboratorio Para a Investigação Integrativa e Translacional em Saude Populacional (ITR), 4200-450 Porto, Portugal; 4 Serviço de Imunoalergologia, Centro Hospitalar São João, 4200-319 Porto, Portugal; 5 Faculdade de Ciências da Nutrição e Alimentação da Universidade do Porto, 4150-4180 Porto, Portugal

**Keywords:** diet, miRNAs, asthma, children, exhaled breath condensate, PRAL, NEAP

## Abstract

Individual nutrients and bioactive compounds have been implicated in the expression of microRNAs (miRNAs), which are related to inflammation and asthma. However, evidence about the impact of diet is scarce. Therefore, we aimed to assess the association between dietary acid load and asthma-related miRNA in the exhaled breath condensate (EBC) of school-aged children. This cross-sectional analysis included 150 participants aged 7 to 12 years (52% girls) from a nested case–control study, which randomly selected 186 children attending 71 classrooms from 20 public schools located in city of Porto, Portugal. Dietary data were collected by one 24 h-recall questionnaire. Dietary acid load was assessed using the potential renal acid load (PRAL) and net endogenous acid production (NEAP) scores. Based on previous studies, eleven asthma-related miRNAs were chosen and analyzed in EBC by reverse transcription-quantitative real-time PCR. PRAL, NEAP and miRNAs were categorized as high or low according to the median. Logistic regression models were performed to assess the association between dietary acid load scores and miRNAs. Children in high dietary acid load groups (PRAL ≥ 14.43 and NEAP ≥ 55.79 mEq/day) have significantly increased odds of having high miR-133a-3p levels. In conclusion, higher dietary acid loads possibly modulate asthma-related miRNAs of school-aged children.

## 1. Introduction

The increased prevalence of asthma from the middle of the 20th century has led to the hypothesis that changes in lifestyle and environmental exposures could be the causes [1]. Among lifestyle changes, dietary habits have shifted and, presently, most populations follow a Western dietary pattern, which is characterized by a high intake of processed meats, refined grains and sugary foods, and low intake of plant-based foods [2]. The lower intake and diversity of fruits and vegetables have been associated with asthma in children [3,4]. In fact, these foods are important sources of dietary fiber, micronutrients and phytochemicals, which participate in antioxidant and anti-inflammatory metabolic activities, possibly modulating asthma development or progression. Therefore, the dietary balance between base-inducing (e.g., fruits and vegetables) and acid-inducing foods (e.g., eggs, cheese, and cereal grains) may be associated with childhood asthma [5].

Nonetheless, although a growing body of evidence suggests a protective effect of base-inducing foods against asthma, little is known of whether their action is mediated through epigenetics [6]. Experimental models showed after fish oil [7] or EPA and DHA exposure [8] a decreased expression of microRNAs (miRNAs), which have been also measured in EBC from individuals and associated with asthma [9,10]. Accordingly, a study conducted on healthy individuals who consumed 30 g/day of almonds and nuts, over 8 weeks, found that the expression of 11 plasma asthma-related miRNA levels was modified [11]. Other experimental studies showed that bioactive compounds, such as quercetin and isorhamnetin treatment of macrophages, downregulated the levels of miRNAs [12] involved in inflammation and asthma [9,10].

Most of the available studies evaluating the influence of dietary components on the modification of gene expression due to epigenetic processes related to microRNAs were performed in vitro and on animal models [13]. Moreover, these studies have been evaluating the effects of dietary components with anti-inflammatory and antioxidant properties. Regardless of specific food intake, dietary components are not ingested alone and may exhibit pleiotropic and synergic effects within each food and between foods when consumed together [14]. Diet composition influences acid–base balance by providing acid or base precursors, whereas lungs play an important role in systemic pH and acid–base regulation [5]. Moreover, our group previously demonstrated that higher dietary acid loads, obtained through a higher intake of animal and lower fruits and vegetables, increased the odds of having asthma in overweight children [15].

In this context, it is plausible that increased dietary acid load might influence miRNA levels related to asthma, providing new insights on the mechanism that explain how diet can modulate inflammation and asthma through epigenetics. Therefore, this study aimed to assess the association between dietary acid load and asthma-related miRNAs in the exhaled breath condensate (EBC) of school-aged children.

## 2. Materials and Methods

### 2.1. Study Design and Participants

We conducted a cross-sectional study conducted from January to April 2014 and October 2014 to January 2015, including students attending 71 classrooms from 20 public primary schools located in Porto Municipality, Portugal.

A total of 1602 children aged 7 to 12 years were invited to participate [16], where 686 did not provide informed consent (*n* = 916), while 58 refused to perform clinical procedures (*n* = 858). Those whose EBC volume was below 400 µL (*n* = 269) were excluded. Two hundred EBC samples were selected for the analysis, including all the participants with asthma (*n* = 74) and a random sample of participants without asthma (*n* = 126) stratified according to BMI [9]. Participants whose EBC samples did not present signal for the control miRNA (*n* = 10) or have quantification cycles above 45 (*n* = 4) as well as those without nutritional data available (*n* = 36) were excluded. At the end, a final sample of 150 children was considered (52 with asthma), whose baseline characteristics are described in Table 1.

### 2.2. 24-Hour-Recall Questionnaire

Dietary intake was assessed through the interviewer-administered 24-h-recall questionnaire, where a detailed collection of foods and beverages consumed in the previous 24 h, including quantities, brands and cooking methods, was performed according to standard procedure [17]. A single questionnaire was applied to the participants, without parents and using a photograph atlas to estimate portion sizes. The software Food Processor^®^ (ESHA Research, Salem, OR, USA) was used to estimate nutritional data. To identify under-reporters, Goldberg cut-offs were used as a direct comparison of energy intake to energy expenditure [18]. Goldberg cut-off values were applied to exclude under-reporters based on physical activity level and compared with the ratio of energy intake to basal metabolic rate. Basal metabolic rate was calculated using the Schofield equations for children based on age, gender, height and weight [19].

### 2.3. Assessment of Dietary Acid Load Scores

The intake of macro- and micronutrients was corrected for total energy intake by normalizing their intakes to a daily intake of 2136 kcal by regression analysis of the residual method [20].

PRAL [21] and NEAP [22] scores were used to assess dietary acid load and were estimated based on the following algorithms [23]:

(1) PRAL (mEq/day) = 0.49 × protein intake (g/day) + 0.037 × phosphorus (mg/day) − 0.021 × potassium (mg/day) − 0.013 × calcium (mg/day) − 0.026 × magnesium (mg/day);

(2) NEAP (mEq/day) = [54.5 × protein intake (g/day) ÷ potassium intake (mEq/day)] − 10.2.

A negative PRAL or NEAP score reflects an alkaline-forming potential, while a positive value reflects an acid-forming potential. Two categories for each score were created based on the median: PRAL-low (<14.43) vs. high (≥14.43); NEAP-low (<55.79) vs. high (≥55.79).

### 2.4. Collection of Exhaled Breath Condensate (EBC)

Children were required to breathe at regular tidal volumes and respiratory rates for 10 to 15 min to obtain a volume of at least 600 μL, which is stipulated as the minimal requirement for a valid sample [24]. An exhaled air condensing system was used in order to collect EBC samples (portable Turbo DECCS), which were transferred to sterile tubes and stored at −80 °C until laboratory analysis. Specifically, a volume of 400 μL was required for miRNA assessment according to the manufacturer’s instructions. The different sample volumes are associated with the children’s tidal and minute volumes of the lungs [25].

### 2.5. Assessment of miRNAs from EBC

We performed an a priori selection of eleven miRNAs based on their association with asthma, obesity and chronic inflammation [10,26,27,28,29,30,31,32,33]: let7a-5p, miR21-5p, miR126-3p, miR133a-3p, miR145-5p, miR146a-5p, miR155-5p, miR221-3p, miR328-3p, miR-1248 and miR-423-3p. In fact, obesity has been suggested to increase asthma incidence and prevalence, and change asthma towards a more difficult-to-control phenotype [34], whereas the obese asthma phenotype is complex and multifactorial [35]. It was recently observed that among five obesity-related risk factors examined for causality with childhood asthma, obesity is observationally and genetically associated with asthma [36]. In addition, our group suggested dietary acid load as a new link in the obese-asthma phenotype [15].

A detailed description of the miRNA assessment can be found in [9]. Briefly, we performed an extraction control with UniSp6 RNA from Exiqon (Qiagen, Woburn, MA, USA), which were used to spike EBC samples. Guanidinium thiocyanate–phenol–chloroform extraction was performed to extract total RNA with the Tri Reagent LS (Sigma-Aldrich, St. Louis, MI, USA). The extracted RNA was quality controlled with a Bioanalyzer (Agilent Technologies, Santa Clara, CA, USA) using the Small RNA Kit (Agilent) according to manufacturer’s instructions. Reverse transcription and real-time quantitative polymerase chain reaction (RT-qPCR) was performed with reagents using the Locked Nucleic Acid (LNA™) technology (Exiqon) through a StepOnePlus Real-Time PCR System (Applied Biosystems, Waltham, MA, USA). The extracted RNA was reverse transcribed (10 μL) in duplicate with the Universal cDNA synthesis kit II (Exiqon) and the resulting cDNA was quantified using miRNA-specific LNA oligos and ExiLENT SYBR Green master mix (Exiqon). GenEX software (MultiD Analyses AB, Göteborg, Sweden) was used for data normalization and analysis. The miRNA let7a-5p was used to normalize the data as selected by NormFinder and geNorm.

Children were categorized into two groups—low and high—according to the median for each miRNA; the cut-off values are summarized in Table 1.

### 2.6. Covariates

Lung function and airway reversibility were assessed by spirometry, recorded before and 15 min after the inhalation of 400 µg of salbutamol, according to American Thoracic Society (ATS)/The European Respiratory Society (ERS) guidelines [37]. Positive bronchodilation (+BD) was defined by at least a 12% and over 200 mL increase in forced expiratory volume in 1 s (FEV_1_) after bronchodilation.

A questionnaire based on the International Study of Asthma and Allergies in Childhood (ISAAC) was given to each child’s legal guardian, from which asthma symptoms occurring in the previous 12 months (wheezing, dyspnea, or dry cough) and self-reported asthma diagnosed by a physician (ever asthma) were retrieved. Asthma was defined by +BD or ever asthma with reported asthma symptoms occurring in the previous 12 months [38].

Anthropometry included the measurement of weight (kg), using a digital scale (Tanita™ BC-418 Segmental Body Analyzer), and height (cm), using a portable stadiometer. Body mass index was calculated and computed as per square meters (kg/m^2^), being classified according to age- and sex-specific percentiles defined by the US Centers for Disease Control and Prevention (CDC) [39].

Parental education level is presented as the number of successfully completed years of formal schooling of the parent with their higher education level and categorized into three classes: ≤9 years; ≥10 years and ≤12 years; and >12 years. Child nutritional supplementation was assessed through a positive answer to the question “Has your child taken nutritional supplements (vitamins/minerals) in the past year?”.

### 2.7. Statistical Analyses

The Kolmogorov–Smirnov test was used to check continuous variables for normality. Continuous variables are presented as median (25th–75th percentiles), while categorical ones are described as counts and proportions. Differences between boys and girls were assessed through the Mann-Whitney Test (for continuous variables) and Chi-squared test (for categorical variables).

Logistic regression models were used to assess the association between the PRAL and NEAP scores with miRNAs and the magnitude of the association is presented as an odds ratio (OR) and its respective 95% confidence interval (CI). In model 1, we adjusted for total energy intake and nutritional supplementation. Model 2 was further adjusted for sex, age (year, continuous), BMI, asthma, school, and parental education. PRAL and NEAP scores were evaluated in separate models.

Significant differences were defined with an α-value below 5% (*p*-value < 0.05). The analyses were performed through the SPSS statistical package software v27.0 (IBM, Armonk, NY, USA) and R software.

## 3. Results

The median (25th–75th) scores for PRAL were 14.92 (4.14; 25.67) and 13.35 mEq/day (0.85; 30.53) for girls and boys, respectively, whereas the median scores for NEAP were 56.16 (45.57; 71.70) mEq/day for girls and 55.69 mEq/day (42.11; 74.27) for boys (Table 1). Significant differences were observed for age and TEI between girls and boys (*p*-value < 0.005, Table 1).

Before adjustment, children with higher PRAL scores had significantly higher EBC miR-133a-3p (OR = 2.52; 95% CI 1.30, 4.86, *p*-value = 0.006, Table 2). This association remained significant after controlling for confounders (model 2 OR = 2.83; 95% CI 1.31, 6.11, *p*-value = 0.008, Table 2).

Similarly, both unadjusted and fully adjusted models showed that higher NEAP scores increased the miR-133a-3p in EBC (unadjusted model, OR = 2.82; 95% CI 1.45, 5.46, *p*-value = 0.002; model 2, OR = 3.00; 95% CI 1.37, 6.60, *p*-value = 0.006, Table 2).

Children with higher NEAP scores were less likely to have higher EBC miR-155-5p (unadjusted model, OR = 0.41; 95% CI 0.19, 0.89, *p*-value = 0.024), even after adjustment for total energy intake and nutritional supplementation (model 1, OR = 0.30; 95% CI 0.12, 0.73, *p*-value = 0.008, Table 2). Nonetheless, after additional adjustment for sex, age, BMI, asthma, and parental education, the association did not remain significant (model 2, OR = 0.41; 95% CI 0.15, 1.10, *p*-value = 0.076, Table 2).

## 4. Discussion

The present study found that high dietary acid loads groups were significantly associated with higher EBC miR-133-3p, which was associated with asthma defined by medical diagnosis with asthma symptoms or +BD in our participants [9]. On the other hand, children in the high NEAP score group additionally showed a tendency to be less likely to have high EBC miR-155-5p, which was negatively associated with symptomatic asthma [9]. This is the first study to assess the effects of diet on exhaled breath condensate miRNAs associated with asthma in children, demonstrating that higher NEAP and PRAL scores might upregulate the expression of miRNAs related to asthma.

Our participants showed to have a diet characterized by an excess of acidifying nutrients, which is in accordance with previous studies [40,41]. Although we did not address the food groups that had the highest acidity and alkalinity impact on children’s dietary acid load, previous studies found differences in these food groups’ contributions, suggesting that different food group aggregations and dietary habits might explain the disparities in the findings [40]. Nonetheless, sulfur amino acids that are higher in animal proteins, nuts and cereals, and phosphorus, which is provided by meat (and meat products) and dairy, might increase dietary acid loads, whereas potassium and magnesium, which are characteristic of plant-based foods, such as fruits and vegetables, and calcium, which is provided by plant-based foods and dairy, decrease dietary acid loads [40,41]. Despite food chemical composition, other factors such as the intestinal absorption rates of specific nutrients, metabolic production of sulfate from sulfur amino acids, the grade of dissociation of phosphorus at the physiological pH, and the ionic valence of calcium and magnesium have an important impact on acid–base balance [42].

Diets following a Western pattern with high acid loads (lower consumptions of vegetables and fruits and high intakes of animal products) might lead to an overproduction and accretion of anions that are not metabolized and might cause a diet-derived metabolic acidosis that will induce compensatory mechanisms, resulting in metabolic consequences and pathologies [43]. However, as proposed by Cunha et al., under normal conditions, pH is maintained within a physiological range since there is excretion of sodium salts from nonvolatile acids by the kidneys and carbon dioxide by the lungs, whereas the intake of alkali precursors foods that will be absorbed in the gastro-intestinal track might neutralize the overload of hydrogen [5]. Accordingly, a previous study conducted in our participants showed a positive and significant association between dietary acid load and asthma in children with overweight/obesity [15].

The present study found that school-aged children in the high dietary acid load group, presumably consuming more animal protein and animal-based products and fewer vegetables and fruits, had significantly increase miR-133a-3p in their airways, whereas a decreased tendency was observed for miR-155-5p in EBC. These results are in accordance with several other studies showing that dietary components might induce epigenetic modifications, including altering miRNAs levels [44]. Specifically, quercetin, which is present in plant-based food with antioxidant and anti-inflammatory properties, was shown to have an impact on miR-155 in experimental studies in animals [45]. These studies demonstrated that a downregulation of its expression resulted in the inhibition of the NF- κB, consequently having an anti-inflammatory effect [12]. Similarly, both in vitro and in vivo studies have showed that vitamin D supplementation decreased NF-κB signaling, resulting in a downregulation of the miR-155 [46,47]. Although the evidence regarding miR-133 is still scarce, an animal model of insulin resistance induced by a high-fat diet showed that, after six weeks, there was an increase in miR-133a in the Western diet group [48].

Several miRNAs have been associated with different pathophysiological features of asthma, including immune development and differentiation, airway inflammation and airway hyper-responsiveness [49]. In fact, it was demonstrated that both EBC miR-133a and miR-155 were significantly decreased in adult participants with asthma compared to a healthy group [10]. Nonetheless, a previous study conducted on the participants from the present study revealed that EBC miR-155-5p was significantly decreased in children with symptomatic asthma, whereas EBC miR-133a-3p was significantly increased in children with asthma [9]. The imbalance of miRNAs involved in the regulation of Th2 cytokine receptors could have implications on the expression and signaling of Th2 cytokines. Specifically, miR-155 in mild asthmatics seems to suppress cytokine expression induced by IL-13 [50], while it is capable of regulating Th2 inflammation through a downregulation of the secretion of IL-4, IL-5 and IL-13 by Th2 cells [51]. On the other hand, it is possible that miR-133a-3p will be induced in immune cells that are stimulated by pro-inflammatory cytokines, such as TNF-α [9], while it seems to be important in the regulation of IL-13, suggesting that deregulation of this pathway could have an impact on the expression and signaling of Th2 cytokines [10].

There is a paucity of observational studies conducted on children and assessing the effect of diet and dietary components on miRNAs related to asthma. Despite the methodological differences regarding research design, age group, sample size, dietary data assessment and selected asthma definitions that might explain different findings among studies, previous results taken together with the findings from this study suggest that diets with higher acid loads might be involved in miRNA expression modulation, being a possible mechanistic link by which diet regulates physiological pathways in asthma.

Our study has some methodological considerations. Considering the cross-sectional design, it is not possible to establish causality. However, there was a detailed collection of data, by the same research team, which assured a relative unbiased estimate of outcomes prevalence. The collection of EBC is simple, safe, noninvasive and highly repeatable and the technology employed is ideal for miRNA profiling. Moreover, a high number of EBC samples were analyzed. Nevertheless, miRNAs were selected based on previous studies that show associations with asthma and obesity [10,26,27,28,29,30,31,32,33]. The protocol was also adjusted to optimize the assessment of miRNAs in our EBC samples. Although the manufacturer’s instructions state a maximum of five cycles above the 40 already allowed and Shi et al. also used 45 cycles to assess miRNAs in EBC samples [52], this number of cycles might have introduced false positives. In addition, some epigenetic changes can last for many years while others can happen within days and certain associations may differ by age [35]. Other limitations are related to the lack of correction for multiple testing. Our findings may also lack external validity as our observations may not be generalizable to different age groups. However, our participants were aged 7 to 12 years, and both diet and EBC collection were performed on the same day.

The dietary data collected might be affected by a recall bias, especially since children’s self-reports of diet are more likely to contain errors due to limited knowledge about food and memory [53]. Nevertheless, although assessing food intake is a difficult task, particularly in children, they may find it easiest to recall the most recent foods consumed, providing detailed data about food preparation methods, ingredients used in mixed dishes, the brand name of commercial products, and the common size containers. In addition, to avoid misreporting dietary consumption, and keeping in mind that portion size is difficult to estimate accurately, 24 h-recall questionnaires were administered by nutritionists and trained interviewers, using photographs and food models to quantify portion sizes, and probing information from children without suggesting responses [54]. Moreover, the 24 h-recall might be more suitable when one wishes to determine the typical dietary intake of large groups of subjects [55].

Although multiple recalls are preferred to report the habitual intake of an individual [56], since one single day does not represent usual intake, 24 h-recall questionnaires can estimate the current diet, without inducing changes in children’s dietary behaviors due to the demanding task of recording or knowing that diet is being assessed [57]. Although children were asked to recall all the foods and drinks consumed in the previous day, a more demanding cognitive task such as comparing their intake of foods in the last 24 h to a typical day was not considered. In addition, it was also assumed that food intake was similar throughout childhood and did not account for seasonality differences.

Dietary acid load was assessed by two methods, including PRAL and NEAP scores, which considered the intake of protein, phosphorous, potassium, calcium, and magnesium, being a validated tool in children [21]. Nonetheless, PRAL and NEAP scores were estimated based on a self-reported dietary intake. In addition, it is important to notice that lower dietary acid loads are compatible with higher intakes of fatty acids, which may play a key role on inflammatory pathways relevant to the pathophysiology of asthma [58]. Further analysis to explore the association between dietary patterns and PRAL and NEAP scores are needed.

## 5. Conclusions

Our findings showed that higher dietary acid loads might modulate asthma-related miRNAs in the airways of school-aged children, as accessed by EBC, suggesting a novel mechanistic link between diet and asthma. Further studies with a prospective design and in other populations are needed to confirm the potential causality between a higher dietary acid load and asthma epigenetic-related impact.

## Figures and Tables

**Table 1:** Participants’ characteristics.

	Females (*n* = 71)	Males (*n* = 79)	Total (*n* = 150)
Age (years)	9.0 (8.0; 9.0)	9.0 (8.0; 10.0)	9.0 (8.0; 9.0) *
Parental education level (years) ^1^, *n* (%)			
≤9	21 (36.84)	27 (40.30)	48 (32.0)
≥10 and ≤12	17 (29.82)	23 (34.33)	40 (26.7)
>12	19 (33.33)	17 (25.37)	36 (24.0)
Nutritional supplementation ^2^, *n* (%)	12 (16.90)	8 (10.13)	20 (13.3)
BMI categories ^3^, *n* (%)			
Underweight	4 (5.63)	5 (6.33)	9 (6.0)
Normal weight	31 (43.66)	32 (40.51)	63 (42.0)
Overweight	23 (32.39)	17 (21.52)	40 (26.7)
Obese	13 (18.31)	25 (31.65)	38 (25.3)
Asthma ^4^, *n* (%)	27 (38.03)	25 (31.65)	52 (34.7)
Energy (kcal/day)	2011.30 (1719.50; 2395.65)	229.21 (1998.35; 2665.99)	2186.07 (1822.60; 2555.45) **
Nutrient intake			
Protein (g/day)	98.76 (87.42; 106.68)	93.64 (84.91; 105.42)	95.43 (85.26; 108.83)
Phosphorus (mg/day)	1469.09 (1305.62; 1653.26)	1415.49 (1203.78: 1640.03)	1443.84 (1250.23; 1640.03)
Potassium (mg/day)	3187.89 (2557.04; 3791.68)	2969.85 (2589.47; 3502.07)	3155.24 (2577.85; 3675.95)
Magnesium (mg/day)	282.33 (244.43; 310.37)	263.32 (2.32.71; 316.83)	269.41 (239.94; 310.36)
Calcium (mg/day)	1002.81 (667.37; 1251.74)	993.10 (779.56; 1268.17)	998.38 (718.56; 1260.81)
Dietary Acid Load (mEq/day)			
PRAL			
Low (<14.43)	33 (46.48)	42 (53.16)	75 (50.0)
High (≥14.43)	38 (53.52)	37 (46.84)	75 (50.0)
NEAP			
Low (<55.79)	35 (49.30)	40 (50.63)	75 (50.0)
High (≥55.79)	36 (50.70)	39 (50.63)	75 (50.0)
MiRNAs			
miR-21-5p			
Low (<2.79)	35 (49.30)	40 (50.63)	75 (50.0)
High (≥2.79)	36 (50.70)	39 (49.37)	75 (50.0)
miR-126-3p			
Low (<0.0568)	36 (50.70)	39 (49.37)	75 (50.0)
High (≥0.0568)	35 (49.30)	40 (50.63)	75 (50.0)
miR-133a-3p			
Low (<0.0164)	32 (45.07)	43 (54.43)	75 (50.0)
High (≥0.0164)	39 (54.93)	36 (45.57)	75 (50.0)
miR-145-5p			
Low (<0.58)	31 (43.66)	44 (55.70)	75 (50.0)
High (≥0.58)	40 (56.34)	35 (44.30)	75 (50.0)
miR-146a-5p			
Low (<0.000665)	47 (66.20)	45 (56.90)	92 (61.3)
High (≥0.000665)	24 (33.80)	34 (43.04)	58 (38.7)
miR-155-5p			
Low (<0.000119)	53 (74.65)	61 (51.90)	114 (76.0)
High (≥0.000119)	18 (25.35)	18 (22.78)	36 (24.0)
miR-221-3p			
Low (<0.0831)	34 (47.89)	41 (51.90)	75 (50.0)
High (≥0.0831)	37 (52.11)	38 (48.10)	75 (50.0)
miR-328-3p			
Low (<0.50)	33 (46.48)	42 (53.16)	75 (50.0)
High (≥0.50)	38 (53.52)	37 (46.84)	75 (50.0)
miR-423-3p			
Low (<0.00107)	51 (71.83)	49 (62.03)	100 (66.7)
High (≥0.00107)	20 (28.17)	30 (37.97)	50 (33.3)

Data are expressed as medians (25th-75th percentile), while categorical ones are described as counts and proportions. * *p*-value < 0.05; ** *p*-value < 0.01 ^1^ Number of successfully completed years of formal schooling; ^2^ child took nutritional supplement in the previous 12 months; ^3^ According to US Centers for Disease Control; ^4^ Positive bronchodilation (defined by at least a 12% and over 200 mL increase in forced expiratory volume in 1 s (FEV1) after bronchodilation) or self-reported asthma diagnosed by a physician with reported asthma symptoms (wheezing, dyspnea, or dry cough) occurring in the previous 12 months. Abbreviations: PRAL: Potential renal acid load. NEAP: net endogenous acid production.

**Table 2:** Association between PRAL and NEAP scores with EBC miRNAs in school-aged children.

		PRAL Score			NEAP Score	
	OR	95% CI	*p*-Value	OR	95% CI	*p*-Value
miR-21-5p						
Unadjusted	0.77	0.40; 1.45	0.766	0.69	0.36; 1.31	0.254
Model 1	0.76	0.39; 1.49	0.418	0.71	0.36; 1.41	0.324
Model 2	0.77	0.36; 1.61	0.480	0.87	0.41; 1.84	0.710
miR-126-3p						
Unadjusted	1.62	0.85; 3.09	0.143	1.62	0.85; 3.09	0.143
Model 1	1.60	0.81; 3.16	0.174	1.50	0.76; 1.00	0.242
Model 2	1.36	0.65; 2.84	0.420	1.36	0.65; 2.86	0.417
miR-133a-3p						
Unadjusted	**2.52**	**1.30; 4.86**	0.006	**2.82**	**1.45; 5.46**	0.002
Model 1	**2.78**	**1.38; 5.57**	0.004	**2.96**	**1.47; 5.96**	0.002
Model 2	**2.83**	**1.31; 6.11**	0.008	**3.00**	**1.37; 6.60**	0.006
miR-145-5p						
Unadjusted	1.06	0.56; 2.00	0.870	1.06	0.56; 2.00	0.870
Model 1	1.12	0.57; 2.21	0.744	1.06	0.54; 2.08	0.877
Model 2	0.92	0.44; 1.95	0.828	1.05	0.49; 2.25	0.893
miR-146a-5p						
Unadjusted	1.57	0.81; 3.05	0.181	1.12	0.58; 2.16	0.737
Model 1	1.28	0.64; 2.58	0.495	0.96	0.47; 1.93	0.897
Model 2	1.56	0.71; 3.41	0.270	1.06	0.48; 2.33	0.880
miR-155-5p						
Unadjusted	0.55	0.26; 1.19	0.129	**0.41**	**0.19; 0.89**	0.024
Model 1	0.46	0.19; 1.08	0.073	**0.30**	**0.12; 0.73**	0.008
Model 2	0.61	0.24; 1.59	0.314	0.41	0.15; 1.10	0.076
miR-221-3p						
Unadjusted	0.77	0.40; 1.45	0.415	0.77	0.40; 1.45	0.415
Model 1	0.77	0.39; 1.51	0.450	0.73	0.37; 1.43	0.360
Model 2	0.78	0.37; 1.63	0.505	0.77	0.37; 1.63	0.501
miR-328-3p						
Unadjusted	1.06	0.56; 2.00	0.870	1.31	0.69; 2.48	0.415
Model 1	1.08	0.55; 2.11	0.834	1.27	0.65; 2.51	0.487
Model 2	1.08	0.51; 2.26	0.848	1.26	0.59; 2.67	0.553
miR-423-3p						
Unadjusted	1.00	0.51; 1.97	0.999	0.70	0.35; 1.38	0.300
Model 1	0.87	0.42; 1.79	0.708	0.63	0.30; 1.31	0.216
Model 2	0.88	0.39; 1.99	0.762	0.61	0.27; 1.39	0.609

Abbreviations: PRAL: Potential renal acid load; NEAP: net endogenous acid production. Model 1, adjusted for total energy intake and nutritional supplementation; model 2, adjusted for the same variables as model 1 plus sex, age (year, continuous), BMI, asthma defined by medical diagnosis with asthma symptoms or +BD, school, and parental education.

## Data Availability

The data presented in this study are available on reasonable request from the corresponding author. The data are not publicly available due to ethical requirements.
